# Inhibitory effects of midostaurin and avapritinib on myeloid progenitors derived from patients with *KIT* D816V positive advanced systemic mastocytosis

**DOI:** 10.1038/s41375-019-0450-8

**Published:** 2019-03-25

**Authors:** Johannes Lübke, Nicole Naumann, Sebastian Kluger, Juliana Schwaab, Georgia Metzgeroth, Erica Evans, Alexandra K. Gardino, Christoph Lengauer, Wolf-Karsten Hofmann, Alice Fabarius, Nicholas C. P. Cross, Andreas Reiter, Mohamad Jawhar

**Affiliations:** 1 Department of Hematology and Oncology, University Medical Centre Mannheim, Mannheim, Germany; 2 Blueprint Medicines Corporation, Cambridge, MA USA; 3 Wessex Regional Genetics Laboratory, Salisbury NHS Foundation Trust, Salisbury, UK; 4 Faculty of Medicine, University of Southampton, Southampton, UK

**Keywords:** Medical research, Haematological diseases

## Abstract

Advanced systemic mastocytosis (advSM) is characterized by the presence of an acquired *KIT* D816V mutation in >90% of patients. In the majority of patients, *KIT* D816V is not only detected in mast cells but also in other hematopoietic lineages. We sought to investigate the effects of the KIT-inhibitors midostaurin and avapritinib on single-cell-derived myeloid progenitor cells using granulocyte-macrophage colony-forming-units of patients with *KIT* D816V positive advSM. Colonies obtained prior to treatment were incubated in vitro with midostaurin (*n* = 10) or avapritinib (*n* = 11) and showed a marked reduction (≥50%) of *KIT* D816V positive colonies in 3/10 (30%) and 7/11 (64%) patient samples, respectively. Three of those 7 (43%) avapritinib responders were resistant to midostaurin in both, in vitro and in vivo. Colonies from four patients with high-risk molecular profile and aggressive clinical course were resistant to both drugs. The in vitro activity of midostaurin strongly correlated with clinical and molecular responses, e.g., relative reduction of *KIT* D816V allele burden and the proportion of *KIT* D816V positive colonies obtained after six months midostaurin-treatment in vivo. We conclude that the colony inhibition assay provides useful information for prediction of responses on midostaurin and that avapritinib has a superior in vitro activity compared to midostaurin.

## Introduction

Systemic mastocytosis (SM) is a rare hematological neoplasm characterized by clonal expansion and multifocal accumulation of neoplastic mast cells affecting various tissues, predominantly bone marrow, skin, and visceral organs. According to the World Health Organisation (WHO) classification, SM can be subclassified into five categories based on the extent of organ infiltration and mast cell related organ damage (indolent SM [ISM], smoldering SM [SSM], SM with an associated hematologic neoplasm [SM-AHN], aggressive SM [ASM], and mast cell leukemia [MCL]) [1–7]. SM-AHN, ASM, and MCL are collectively referred to as advanced SM (advSM), a poor-prognostic disease with a median overall survival (OS) between three and four years [8–12].

In more than 90% of advSM patients, somatic gain-of-function point mutations in *KIT* are detectable, usually the substitution of aspartate (D) to valine (V) at position 816 (*KIT* D816V) in the kinase domain [13, 14]. A majority of patients with *KIT* D816V positive advSM harbor additional somatic mutations, most frequently in *TET2*, *SRSF2*, *ASXL1*, *RUNX1*, *JAK2* or *N*/*KRAS* [10, 15–18]. In advSM patients, the presence of mutations in *SRSF2*, *ASXL1,* and/or *RUNX1* (S/A/R gene panel) confers a strong adverse impact on phenotype, response to midostaurin, progression to more advSM subtypes, and OS [9, 10, 19].

Because of the significance of *KIT* D816V in the pathogenesis of advSM, targeted drugs against the oncogenic mutation have been developed. Assessing the safety and efficacy of midostaurin (PKC-412) in a multicenter, open-label, single-arm phase 2 study (NCT00233454), the multikinase/KIT-inhibitor (IC_50_ of 2.9 nM) has demonstrated an overall response rate (ORR; major + partial response) of 60% per Valent criteria (28% in a separate *post hoc* analysis by the European medicines Agency [EMA] according to the International Working Group-Myeloproliferative Neoplasms Research and Treatment [IWG-MRT] & European Competence Network on Mastocytosis [ECNM] consensus criteria) in advSM patients leading to approval by the US Food and Drug Administration (FDA) and EMA in 2017 [20, 21]. However, validated biomarkers for prediction of response in advSM patients treated with midostaurin are still lacking. Avapritinib (BLU-285), a potent and highly selective *KIT* D816V inhibitor (IC_50_ of 0.27 nM), has shown preclinical activity as well as encouraging results in an open-label, dose-escalation in phase I trial evaluating the safety and antineoplastic activity (NCT02561988) [22–24].

The aim of the present study was to establish an amenable in vitro assay to investigate the inhibitory effects of midostaurin and avapritinib on single-cell-derived myeloid progenitor cells using granulocyte-macrophage colony-forming-units (CFU-GM) of patients with *KIT* D816V advSM and to correlate in vitro colony data with clinical and molecular characteristics at baseline, and response parameters of midostaurin-treated advSM patients in vivo.

## Methods

### Patient characteristics and response criteria

A total of 13 patients with advSM (SM-AHN, *n* = 11; ASM, *n* = 2) were examined. The median age was 67 years (range 48–79). The median OS from time of diagnosis was 33 months (range 13–283). The median bone marrow mast cell infiltration, determined by immunohistochemistry, was 35% (range 20–70) and median serum tryptase level was 140 µg/L (range 33–739). Additional relevant laboratory, clinical, molecular, and cytogenetic parameters including SM-associated disease characteristics at baseline are summarized in Table 1, and for each individual patient in Tables 2 and 3, respectively. Patients were diagnosed and subtyped according to the WHO 2016 classification [1–7]. Various myeloid AHNs were observed (chronic myelomonocytic leukemia, CMML, *n* = 3; myelodysplastic/myeloproliferative neoplasm unclassified, MDS/MPN-U, *n* = 7; MPN with eosinophilia, *n* = 1).

**Table 1:** Summarized clinical, laboratory, histological, and molecular characteristics of 13 *KIT* D816V positive advanced systemic mastocytosis patients prior to treatment based on response pattern in single-cell-derived myeloid progenitor cells (CFU-GM colonies, relative reduction of *KIT* D816V positive colonies), three cohorts were defined: midostaurin + avapritinib responder (cohort #1), midostaurin non-responder + avapritinib-responder (cohort #2), and midostaurin + avapritinib non-responder (cohort #3)

	Initial	Cohort #1	Cohort #2	Cohort #3
Number of patients	13	4	3	4
Age in years; median (range)	67 (48–79)	58 (48–79)	76 (75–78)	64 (61–67)
Male, *n* (%)	11 (85)	3 (75)	3 (100)	3 (75)
C-findings^a^				
C-findings, *n*; median (range)	3 (2–4)	3 (2–4)	3 (2–3)	3 (2–4)
Hemoglobin, g/dL; median (range)	9.9 (7.1–15.0)	10.8 (7.1–15.0)	9.4 (8.8–12.0)	11.7 (9.1–13.9)
< 10 g/dL,* n* (%)	7 (54)	2 (50)	2 (67)	1 (25)
Platelets, ×10^9^ /L; median (range)	110 (29–426)	190 (29–425)	108 (80–315)	117 (47–426)
< 100 × 10^9^ /L, *n* (%)	5 (38)	1 (25)	1 (33)	2 (50)
ANC, ×10^9^ /L; median (range)	7.5 (1.0–60.0)	8.7 (1.7–12.6)	1.3 (1.0–6.1)	16.4 (6.2–60.6)
< 1 × 10^9^ /L, *n* (%)	0 (0)	0 (0)	0 (0)	0 (0)
Alkaline phosphatase, U/L; median (range)	376 (41–707)	204 (41–707)	409 (303–592)	387 (78–632)
> 130 U/L, *n* (%)	11 (85)	3 (75)	3 (100)	3 (75)
Albumin level, g/L; median (range)	34.5 (30.0–43.0)	33.1 (29.5–40.7)	34.5 (33.6–34.5)	34.6 (33.6–42.9)
< 34 g/L, *n* (%)	6 (46)	2 (50)	1 (33.3)	2 (50)
Weight loss (>10% over last 6 months), *n* (%)	8 (62)	4 (100)	1 (33.3)	3 (75)
B-findings				
MC-infiltration in BM biopsy, %, median (range)	35 (20–70)	30 (20–50)	50 (20–60)	20 (20–50)
Serum tryptase level, µg/L; median (range)	140 (33–739)	104 (40 –194)	213 (128–739)	173 (102–225)
Organomegaly^b^, *n* (%)	12 (92)	3 (75)	3 (100)	3 (100)
Other relevant findings				
Leukocytes, × 10^9^/L median (range)	10.8 (2.2–87.0)	12 (3.9–15.4)	3.4 (2.2–8.9)	20.7 (9.1–86.6)
Monocytes, × 10^9^/L median (range)	0.8 (0.2-6.9)	0.5 (0.4–0.6)	0.5 (0.3–1)	1.5 (0.2–6.9)
Eosinophils, × 10^9^/L median (range)	0.4 (0.1–3.6)	0.2 (0.1–0.3)	0.45 (0.1–1.2)	1.5 (1.5–1.5)
* KIT* D816V EAB in PB, %, median (range)	40 (18–55)	27 (18–47)	41 (40–43)	51 (40–55)
Additional mutations besides *KIT* D816V^c^	2 (0–5)	1 (0–1)	2 (2–3)	4 (2–5)

*ANC* absolute neutrophil count, *BM* bone marrow, *EAB* expressed allele burden, *MC* mast cell, *PB* peripheral blood

^a^Non-measurable C-findings (e.g., ascites and osteolytic lesions) were excluded

^b^Organomegaly including hepatomegaly, splenomegaly and/or lymphadenopathy

^c^Additional mutations were detected using targeted sequencing panel to investigate 18 candidate genes

**Table 2:** Patient specific clinical, laboratory, histological, and molecular profile of 13 *KIT* D816V positive advanced systemic mastocytosis patients

#	Age in years	Sex	Type of SM	AHN	A/T	M/E	Karyotype	MC infiltration in BM (%)	Serum tryptase (µg/L)	*KIT* D816V EAB in BM (%)	*SRSF2*	*ASXL1*	*RUNX1*	*TET2*	Other mutations
1	78	M	ASM	MDS/MPN-U	−/−	+/+	−	20	128	45	1	−	1	1	−
2	75	M	ASM	CMML	+/−	+/−	46,XY[25]	50	213	21	1	−	−	1	−
3	79	M	ASM	MDS/MPN-U	+/+	−/−	46,XY[25]	20	68	30	−	−	−	1	−
4	61	M	ASM	MPNeo	−/−	−/+	complex	20	131	44	1	1	−	1	−
5	76	M	MCL	MDS/MPN-U	+/+	−/−	46,XY[22]	60	739	50	1	−	−	−	*IDH2*
6	64	M	ASM	MDS/MPN-U	−/+	+/−	46,XY[25]	50	225	64	1	1	−	−	−
7	57	M	ASM	MDS/MPN-U	+/−	−/−	46,XY[20]	50	140	–^a^	1	−	−	−	−
8	67	F	MCL	CMML	−/−	+/−	46,XX[23]	20	102	58	1	1	−	1	*EZH2*
9	76	M	ASM	CMML	+/+	+/−	46,XY,9qh+[25]	20	33	41	1	1	−	1	−
10	75	M	ASM	MDS/MPN-U	+/−	+/+	46,XY[25]	70	305	–^b^	1	−	−	1	−
11	56	M	ASM	−	−/−	−/−	45,X,-Y[24]	35	194	45	−	−	−	−	−
12	67	M	ASM	MDS/MPN-U	+/+	+/−	46,XY[20]	20	214	42	1	1	1	1	*MPL*
13	48	F	ASM	−	−/−	−/−	46,XX[25]	20	40	22	−	−	−	−	−

*A/T* anemia < 10.0 g/dL (+), > 10.0g/dL (−), platelets < 100 × 10^9^/L (+), > 100 × 10^9^/L (−), *AHN* associated hematologic neoplasm, *ASM* aggressive systemic mastocytosis, *BM* bone marrow,* CMML* chronic myelomonocytic leukemia, *EAB* expressed allele burden, *F* female, *M* male, *MC* mast cell, *MCL* mast cell leukemia, *MDS* myelodysplastic syndrome, *M/E* monocytosis > 1 × 10^9^/L (+), < 1 × 10^9^/L or unknown (−), eosinophilia > 1 × 10^9^/L (+), < 1 × 10^9^/L or unknown (−), *MDS/MPN-U* myelodysplastic/myeloproliferative neoplasm, unclassified, *MPNeo* myeloproliferative neoplasm with eosinophilia

#Patient number

^a^Data not available,* KIT* D816V EAB in peripheral blood (PB) was 43%

^b^Data not available, *KIT* D816V EAB in PB was 33%

**Table 3:** Response data in single-cell-derived myeloid progenitor cells (CFU-GM colonies) on midostaurin and avapritinib in 13 *KIT* D816V positive advanced systemic mastocytosis patients stratified in midostaurin + avapritinib responder (cohort #1), midostaurin non-responder + avapritinib-responder (cohort #2), midostaurin + avapritinib non-responder (cohort #3), and midostaurin responder^f^ (cohort 4) according to relative reduction of *KIT* D816V positive colonies

#	Midostaurin in vivo (months)	Response^a^ (Valent *et al*.) [3]	*KIT* D816V EAB change in PB on midostaurin^b^ (%) (Jawhar *et al*.) [20]	OS from diagnosis (months)	Death (yes/no)	*KIT* D816V positive colonies (%) (prior to treatment)	*KIT* D816V positive colonies (%) (on midostaurin in vivo)^c^	*KIT* D816V positive colonies (%) (on midostaurin in vitro)^d^	*KIT* D816V positive colonies (%) (on avapritinib in vitro)^e^
Cohort #1									
3	6	Yes (MPR)	82 (↓)	42	No	100	40	50	0
7	23	Yes (IR)	43 (↓)	33	No	70	10	−	0
11	13	Yes (IR)	72 (↑)	133	No	80	80	40	10
13	20	Yes (IR)	76 (↓)	283	No	30	10	10	0
Cohort #2									
1	3	No (PD)	0	23	Yes	40	−	60	0
2	3	No (PD)	−	22	Yes	100	−	100	0
5	7	No (PD)	23 (↑)	21	Yes	90	90	90	10
Cohort #3									
4	7	No (PD)	3 (↑)	13	Yes	90	90	90	70
6	6	No (PD)	0	15	Yes	100	100	100	80
8	7	No (PD)	113 (↑)	34	Yes	100	100	100	100
12	11	No (PD)	24 (↓)	20	Yes	95	95	90	100
Cohort #4									
9	31	Yes (MPR)	73 (↓)	54	Yes	90	5	−	−
10	22	Yes (IR)	62 (↓)	46	Yes	100	10	−	−

*CFU-GM* granulocyte-macrophage colony-forming-unit, *EAB* expressed allele burden, *IR* incomplete remission, *MPR* minor partial response, *OS* overall survival, *PB* peripheral blood, *PD* progressive disease

#Patient number

^a^Response according to modified Valent response criteria

^b^*KIT* D816V EAB change from baseline to month six

^c^*KIT* D816V positive colonies from patients on midostaurin at month six

^d^*KIT* D816V positive colonies incubated with midostaurin (600 nM) for two weeks

^e^*KIT* D816V positive colonies incubated with avapritinib (75 nM) for two weeks

^f^Data on avapritinib was not available

The clinical response to treatment was evaluated by measurable C-findings (excluding ascites and osteolytic lesions) according to modified Valent response criteria as previously described [3, 20].

Reference pathologists of the ECNM evaluated all bone marrow biopsies. The study design adhered to the tenets of the Declaration of Helsinki and was approved by the relevant institutional review board of the Medical Faculty of Mannheim, Heidelberg University, as part of the ‘German Registry on Disorders of Eosinophils and Mast Cells’. All patients provided written informed consent.

### Quantitative assessment of *KIT* D816V

Quantitative assessments of the *KIT* D816V expressed alele burden (EAB) were performed using allele-specific quantitative real-time reverse-transcriptase polymerase chain reaction (qRT-PCR) analysis on RNA/complementary DNA as previously described [14].

### Targeted next-generation sequencing (NGS) analysis

Next-Generation Deep Amplicon Sequencing by 454 FLX amplicon chemistry (Roche, Penzberg, Germany) with consistent detection sensitivity of EAB down to 3% was performed in all patients to investigate 18 candidate genes as previously described [15]. The customized sequencing panel targeted the hotspot or complete coding regions of the following 18 genes: *ASXL1*, *CBL*, *ETV6*, *EZH2*, *IDH1*, *IDH2*, *JAK2*, *KRAS*, *NPM1*, *NRAS*, *RUNX1*, *SETBP1*, *SF3B1*, *SRSF2*, *TET2*, *TP53*, *U2AF1*, and *ZRSR2*. The sequential NGS approach is based on library preparation by the Access Array Technology (Fluidigm, San Francisco, CA) and sequencing on the MiSeq Instrument (Illumina, San Diego, CA). Gene mutations were annotated using the reference sequence of the Ensembl Transcript ID (Ensembl release 85: July 2016).

### CFU-GM colony assay

**Fig. 1:**
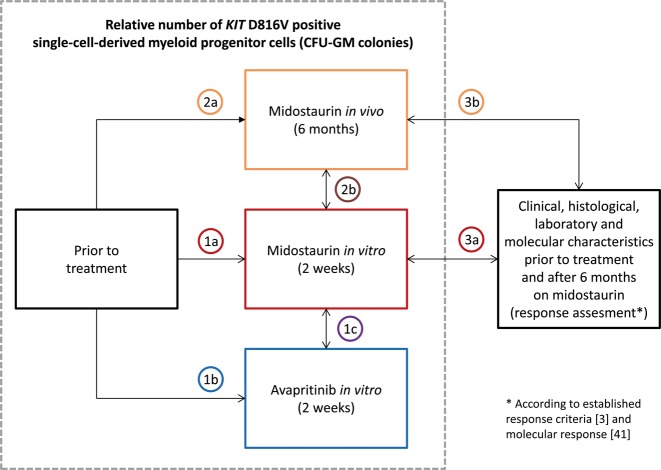
This figure outlines the design of the study. Comparison (->) or correlation (<->) of the relative reduction of *KIT* D816V positive single-cell-derived myeloid progenitor cells (CFU-GM colonies) between: prior to treatment versus midostaurin in vitro (1a) or avapritinib in vitro (1b), midostaurin in vitro versus avapritinib in vitro (1c), prior to treatment versus midostaurin in vivo (2a), midostaurin in vivo versus midostaurin in vitro (2b), and patients profile (including clinical, laboratory, histological, and molecular data) and established response assessment [3, 41] (after six month midostaurin treatment) versus midostaurin in vitro (3a) and in vivo (3b) assay. CFU-GM granulocyte-macrophage colony-forming-unit

The CFU-GM colony assay is an in vitro assay based on primary bone marrow mononuclear cells using semi-solid methylcellulose (0.9%) matrix supplemented with 30% fetal bovine serum albumin (FBS), 1% BS albumin, 0.1 M 2-mercaptoethanol and recombinant human GM-CSF (100 ng/ml; MethoCult, StemCell Technologies, Cologne, Germany) in 35 mm Petri-dishes. The cells (1 × 10^5^ cells in 1 mL MethoCult) were incubated at 37 °C in a humidified atmosphere with 5% CO_2_ until colonies appeared after 10–14 days. Per colony, 100–300 cells were diluted in phosphate-buffered saline. Based on previous publications and for proof-of-principle, we incubated treatment-naive CFU-GM colonies with 100 nM to 1 µM of midostaurin and 22 nM to 90 nM of avapritinib, respectively. Based on the obtained data from these assays (maintenance of colony growth in combination with optimum decreasing of *KIT* D816V positive CFU-GM colonies), we performed our experiments with 600 nM midostaurin and 75 nM avapritinib, respectively [25–27]. Figure 1 outlines an overview on the various colony assays.

### Genotyping of CFU-GM

Whole-genome amplification (REPLI-g, Qiagen, Hilden, Germany) was performed to determine the mutational status of single-cell-derived CFU-GM colonies (mean colonies per assay per patient, *n* = 15; range 10–30, at least 10 colonies were evaluated). Sanger sequencing for mutation validation of *KIT* D816V and additional mutations was performed after PCR amplification of the relevant region. CFU-GM colonies are expected to be either positive (50% in case of heterozygosity, 100% in case of homozygosity) or negative for any mutation since they are derived from a single myeloid progenitor cell.

### Cytogenetic analysis

For cytogenetic analysis, at least 20 Giemsa-banded bone marrow metaphases cultured for 24 h and/or 48 h were prepared as previously described, analyzed by G-/R-banding technique and interpreted according to the International System for Human Cytogenetic Nomenclature [28, 29].

### Statistical analysis

All statistical analyses considered clinical and laboratory parameters as well as experimental data obtained at the time of midostaurin initiation and after six months treatment (in vivo). Pearson’s correlation coefficient was used to compare the change of *KIT* D816V positive colonies in vitro after two weeks incubation with midostaurin and avapritinib and in vivo after six months midostaurin-treatment. The phi coefficient was used to evaluate the association between response according to the mutational status and the *KIT* D816V EAB in peripheral blood and response to midostaurin in vitro/in vivo. A paired *t*-test was used to compare the relative reduction in the proportion of *KIT* D816V positive colonies from baseline to in vitrocolonies incubated with midostaurin and avapritinib. OS was defined as the time between diagnosis and the date of death or last contact. *P* values < 0.05 (two-sided) were considered significant. GraphPad Prism Software (version 5, GraphPad, La Jolla, CA, USA) and SPSS (version 21.0.0, IBM Cooperation, Armonk, NY) were used for statistical analysis.

## Results

### Molecular characteristics prior to treatment

In addition to *KIT* D816V in all 13 cases, we identified somatic mutations in seven different genes: *SRSF2* (*n* = 10), *ASXL1* (*n* = 5), *RUNX1* (*n* = 2), *TET2* (*n* = 8), *IDH2* (*n* = 1), *EZH2* (*n* = 1) and *MPL* (*n* = 1) (Table 2). Eleven of 13 (85%) patients showed 1 (*n* = 2), 2 (*n* = 4), 3 (*n* = 3), 4 (*n* = 1) or 5 (*n* = 1) additional somatic mutation(s). At least one mutation in the S/A/R gene panel was identified in 10/13 cases (77%). No additional mutations were found in two patients. Two of 13 (15%) patients presented with an aberrant karyotype (Table 2).

### In vitro efficacy of midostaurin and avapritinib

**Fig. 2:**
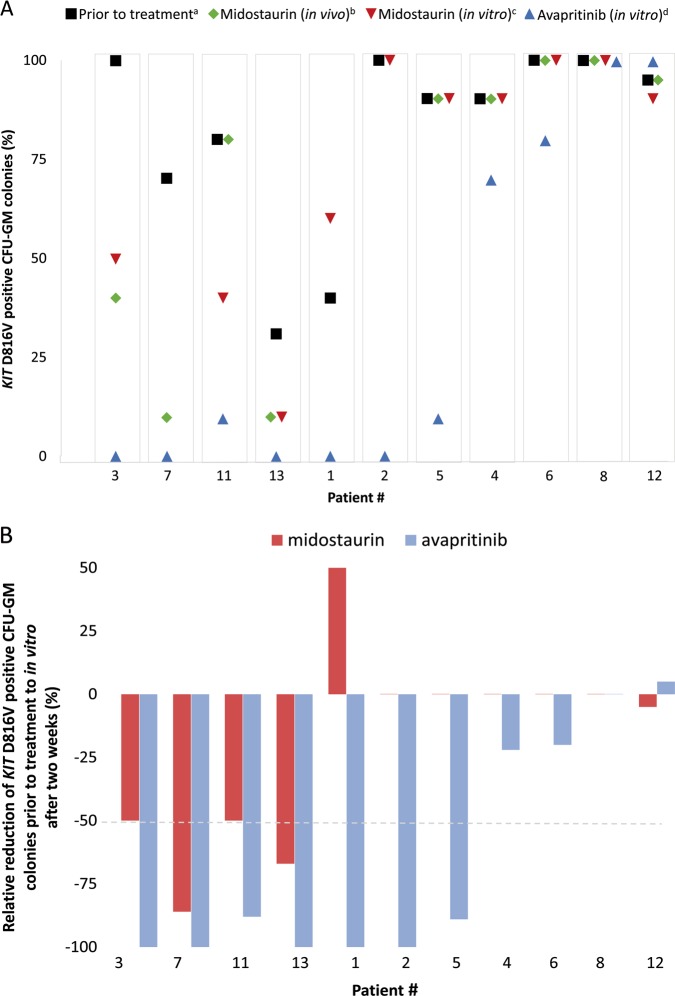
**a** Summarizes in vivo and in vitro data regarding the proportion of *KIT* D816V positive single-cell-derived myeloid progenitor cells (CFU-GM colonies) for each patient: ^a^prior to treatment, ^b^colonies after six months midostaurin-treatment in vivo, ^c^colonies incubated in vitro with midostaurin for two weeks, ^d^colonies incubated in vitro with avapritinib for two weeks. CFU-GM, granulocyte-macrophage colony-forming-unit. **b** Relative reduction in the proportion of *KIT* D816V positive colonies from baseline (prior to treatment) to in vitro colonies incubated with midostaurin (red) and avapritinib (blue). In patient #7, midostaurin in vivo data was used (in vitro data not available). Patient order is based on response pattern (responder: at least 50% relative reduction of *KIT* D816V positive colonies): midostaurin + avapritinib responder (cohort #1; patient #3, #7, #11, #13), midostaurin non-responder + avapritinib-responder (cohort #2; patient #1, #2, #5), and midostaurin + avapritinib non-responder (cohort #3; patient #4, #6, #8, #12). CFU-GM granulocyte-macrophage colony-forming-unit

To evaluate the activity of midostaurin and avapritinib against advSM in vitro, we grew CFU-GM colonies from patients in the presence or absence of each drug. For all 13 cases, a median of 90% (range 30–100) of colonies obtained prior to treatment and grown in the absence of either midostaurin or avaprinitib tested positive for *KIT* D816V (Table 3). When treated with midostaurin (mean number of colonies per assay and patient, *n* = 10, data available in 10/13 cases) or avapritinib (mean number of colonies per assay and patient, *n* = 10, data available in 11/13 cases), a median of 90% and 10% of colonies (*p* = 0.0102, Fig. 2b), respectively, were still *KIT* D816V positive, with 3/10 (30%, #3, #11, #13) and 7/11 patients (64%, #1, #2, #3, #5, #7, #11, #13), respectively, showing a ≥ 50% reduction (responder) of *KIT* D816V positive colonies (Table 3, Fig. 2a, b). Three of those seven (43%) avapritinib responders (#1, #2, #5) were resistant to midostaurin while four avapritinib non-responders were also resistant to midostaurin (#4, #6, #8, #12).

### Various response patterns of colonies on midostaurin and avapritinib

Based on response pattern of colonies (relative reduction of *KIT* D816V positive colonies), three cohorts were defined: midostaurin and avapritinib responder (cohort #1, *n* = 4), midostaurin non-responder and avapritinib-responder (cohort #2, *n* = 3), and midostaurin or avapritinib non-responder (cohort #3, *n* = 4). The comparison between those cohorts reveals no significant differences regarding pure mast cell burden including mast cell bone marrow infiltration (28, 50 and 20%; *p* = 0.2909) and serum tryptase (104, 213, and 173 µg/L; *p* = 0.1912), but significant differences regarding disease burden, median *KIT* D816V EAB (30, 45, and 51%; *p* = 0.0411) and number of S/A/R mutation(s) (0–1, ≥ 2 and ≥ 2; *p* = 0.029). No significant differences were seen regarding the various subtypes of advSM or karyotype (Tables 1–3).

### Effect of midostaurin and avapritinb on additional somatic mutations

Colonies (mean colonies per assay per patient, *n* = 10) were tested for somatic mutations that had previously been identified by bulk analysis. Neither midostaurin nor avapritinib had an inhibitory effect in terms of relative reduction of colonies positive for additional somatic mutations (patients #4: *SRSF2*, *ASXL1*, *TET2*; #5: *SRSF2*, *IDH2*; #7: *SRSF2*; #8: *SRSF2*, *ASXL1*, *TET2*, *EZH2;* #9: *SRSF2*, *ASXL1*, *TET2*; #10: *SRSF2*, *TET2*). In addition, longitudinal data on additional somatic mutations were available in five patients after six months in vivo treatment with midostaurin. In patient #4, a new *NPM1* mutation emerged after 6 months while in patient #5 the variant allele frequency of the *IDH2* mutation raised from 20 to 49%.

### Overall correlation between colony inhibitory assays and clinical/molecular characteristics

**Fig. 3:**
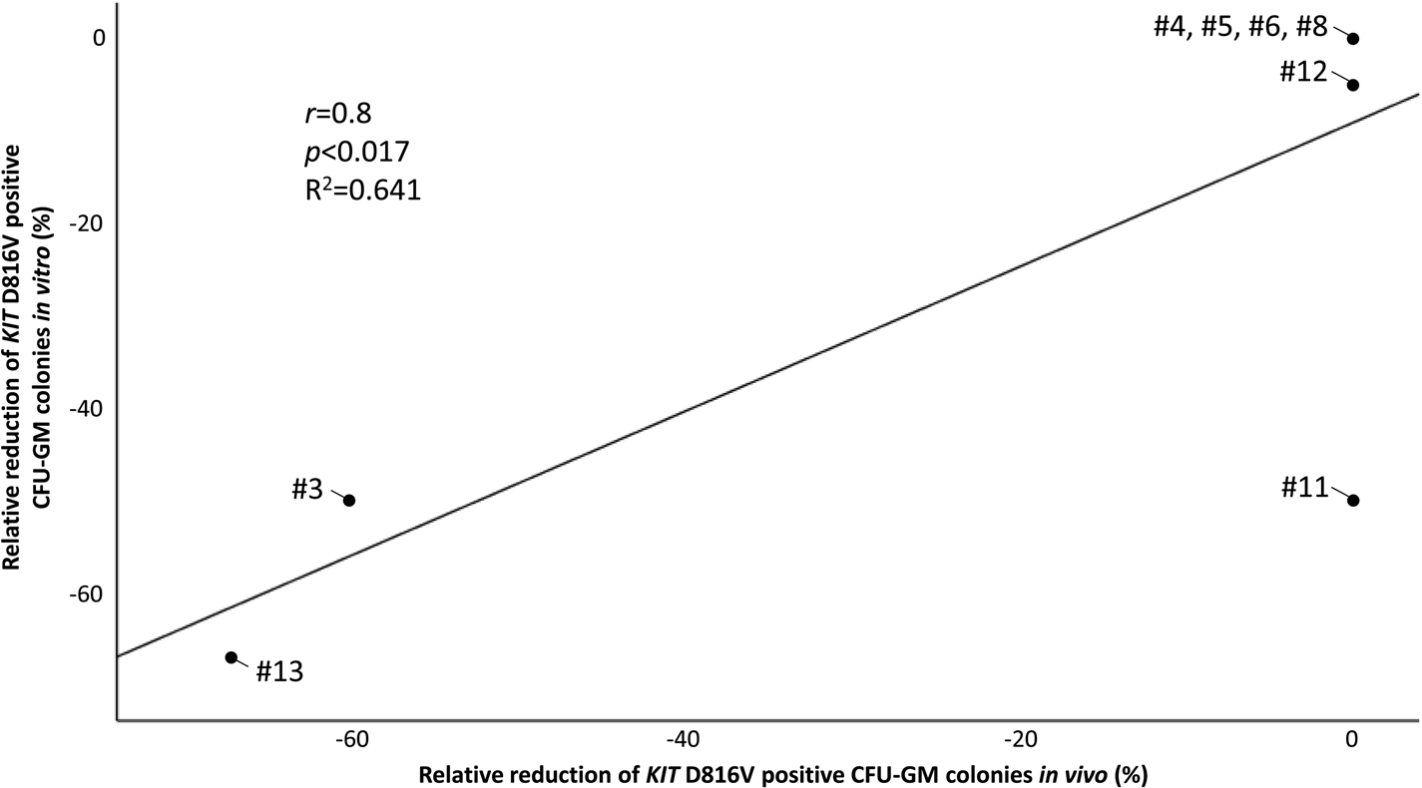
Correlation between the relative reduction of *KIT* D816V positive single-cell-derived myeloid progenitor cells (CFU-GM colonies, in comparison to proportion of *KIT* D816V positive colonies obtained prior to treatment) after in vitro incubation with midostaurin (2 weeks) and in vivo midostaurin treatment (6 months). CFU-GM, granulocyte-macrophage colony-forming-unit

The comparison between colonies obtained prior to treatment and after 6 months treatment of patients (*n* = 11) with midostaurin (in vivo) revealed that 5/11 (45%) patients (#3, #7, #9, #10, #13, Table 3, Fig. 2a) had a ≥ 50% reduction of *KIT* D816V positive colonies. Overall, a significant correlation was observed between the relative reduction of *KIT* D816V positive colonies in vitro and (a) the relative reduction of *KIT* D816V positive colonies after 6 months midostaurin in vivo (*r* = 0.8, *p* < 0.017, *R*^2^ = 0.641, Fig. 3), (b) the absence of any mutation in the S/A/R gene panel (*p* < 0.033) and (c) clinical (according to modified Valent response criteria) and molecular (reduction of *KIT* D816V EAB in peripheral blood ≥ 25%, *p* < 0.003, Tables 4a, b) response.

**Table 4a:** Correlation between response according to *KIT* D816V expressed allele burden and response to midostaurin in vitro

		Response to midostaurin in vitro^b,c^		All
		No	Yes	
Response according to *KIT* D816V EAB in PB^a^	No	6	1	7
	Yes	0	5	5
	All	6	6	12

*EAB* expressed allele burden, *PB* peripheral blood

^a^Response defined as reduction of the *KIT* D816V EAB in PB ≥ 25% after six months [20]

^b^Response defined as reduction of *KIT* D816V positive colonies ≥ 50% after two weeks in vitro

^c^In three cases, in vivo data was used for statistical analysis because in vitro data was not available

**Table 4b:** Correlation between expected response according to mutation(s) in the *SRSF2*, *ASXL1*, and *RUNX1* (S/A/R) gene panel and response to midostaurin in vitro

		Response to midostaurin in vitro^a,b^		All
		No	Yes	
S/A/R mutational status	0	0	3	3
	≥ 1	7	3	10
	All	7	6	13

^a^Response defined as reduction of *KIT* D816V positive colonies ≥ 50% after two weeks in vitro

^b^In three cases in vivo data was used for statistical analysis because in vitro data was not available

## Discussion

In the vast majority of patients with advSM, the *KIT* D816V mutation is not only present in the mastcell lineage but also in multiple hematopoietic lineages (including the AHN compartment) [30–32]. The *KIT* D816V mutation can also be identified in CFU-GM colonies generated from myeloid progenitors [31] and recent data have highlighted the usefulness of these colonies for obtaining a more thorough insight into the clonal architecture of SM and other multimutated myeloid neoplasms [33–39].

In addition to improvement of C-findings, the assessment of responses is based on the relative reduction of mast cell burden, e.g., mast cell infiltration in bone marrow and serum tryptase [20, 40]. However, this approach may not be sufficient to assess response in the non-mast cell (AHN) compartment of SM-AHN. In this respect, recent data have highlighted the importance and potential superiority of changes of the *KIT* D816V EAB as it represents in fact both compartments [41]. We therefore sought to assess the inhibitory effects of midostaurin and avapritinib on primary myeloid progenitor cells derived from *KIT* D816V positive advSM patients.

After two weeks incubation with midostaurin and avapritinib in vitro, the relative reduction of *KIT* D816V colonies was superior on avapritinib, including number of patients and depth of response. Of interest, three midostaurin non-responders had a significant response to avapritinib, while four avapritinib non-responders showed neither a response on midostaurin. These four patients were characterized by a relatively low mast cell burden with regard to mast cell infiltration in bone marrow histology and serum tryptase level but a very high *KIT* D816V EAB (representing disease burden of both SM and AHN) and a poor-prognostic molecular risk profile with ≥ 2 mutations in the S/A/R gene panel. This data indicates that the *KIT* D816V EAB as marker for overall disease burden and the presence of additional somatic mutations in the S/A/R gene panel may be more important for prediction of response and resistance as the pure mast cell burden (Tables 1 and 3, Fig. 2a, b).

The efficacy and safety of the highly selective *KIT* -inhibitor avapritinib in patients with advSM is currently being evaluated in an open-label, single-arm phase 2 study (NCT03580655). In an initial dose-escalation phase 1 study (NCT02561988), avapritinib demonstrated an ORR of 83% per IWG-MRT & ECNM consensus criteria in 29 evaluable patients. Consistent with our in vitro data, a therapeutic benefit of avapritinib was also observed in several patients with primary or secondary resistance on midostaurin [21, 22, 24, 42].

On midostaurin, the relative reduction of *KIT* D816V positive colonies after two weeks incubation in vitro was fully paralleled by the relative reduction of *KIT* D816V positive colonies after 6 months therapeutic treatment (Fig. 3) and by the pattern of clinical response and resistance (Table 3). The in vitro responses were strongly associated with absence of mutations in the S/A/R gene panel (*p* < 0.033) and reduction of the *KIT* D816V EAB ≥ 25% at month six (*p* < 0.003), parameters which were recently reported to be most predictive for response to treatment and favorable outcome (Tables 4a, b) [41]. This data therefore proves the hypothesis that midostaurin is not only able to target the mast cell compartment but also the *KIT* D816V positive AHN.

Disparate mechanisms may confer to resistance to midostaurin and avapritinib. We recently revealed the negative impact of mutations in the S/A/R gene panel on phenotype, response rates, resistance, early or late progression and consequently survival in midostaurin-treated patients suggesting primary resistance and/or outgrowth of a multimutated and clinically aggressive *KIT* D816V positive clone [9, 15, 41]. We now could also demonstrate that neither midostaurin nor avapritinib had an effect on the multimutated *KIT* D816V negative compartment, which may lead to KIT independent resistance and progression, e.g., secondary *KIT* D816 negative acute myeloid leukemia [43]. Other potential mechanisms of resistance to midostaurin and avapritinib may be unveiled in ongoing and upcoming clinical trials.

In conclusion, midostaurin is not only able to target the mast cell compartment but also the *KIT* D816V positive AHN while it may not overcome the adverse effect of high molecular risk mutations (S/A/R gene panel). The in vitro inhibition assay could be considered as a prognostic tool to predict the in vivo response to midostaurin (and potentially also to avapritinib) in patients with advSM. The highly selective *KIT* -inhibitor avapritinib has significant in vitro activity against *KIT* D816V, even in midostaurin non-responders. It will therefore be most interesting to extend this exploratory analysis to a larger cohort of midostaurin-treated patients but also to avapritinib-treated patients with or without prior midostaurin treatment. This assay may then help to determine the choice and sequence of available treatment options, e.g., in terms of the potential sequential use of KIT-inhibitors and alternative treatment options in non-responders including (intensive) chemotherapy and potentially early allogeneic stem cell transplantation [4, 5, 20, 44].
